# The chloroform fraction of *Dracontium spruceanum* modulates gene expression in gastric cancer stem cells

**DOI:** 10.1038/s41598-025-15172-9

**Published:** 2025-08-27

**Authors:** Salyoc Tapia-Rojas, José Amiel-Pérez, Alejandro Fukusaki-Yoshizawa, Obert Marín-Sánchez, Ana Mayanga-Herrera

**Affiliations:** 1 Cell Culture and Immunology Lab, Universidad Científica del Sur, Antigua Panamericana Sur Km 19, 15067 Lima, Peru; 2 Cancer and Stem Cells Research Group, Universidad Científica del Sur, Antigua Panamericana Sur Km 19, 15067 Lima, Peru; 3 Laboratorio de Investigación en Química y Bioquímica de Productos Naturales, Universidad Científica del Sur, Antigua Panamericana Sur Km 19, 15067 Lima, Peru; 4 Academic Department of Medical Microbiology, Faculty of Medicine, Universidad Nacional Mayor de San Marcos, 15081 Lima, Peru

**Keywords:** RNA, Biomarkers, Drug development, Preclinical research

## Abstract

Gastric cancer is the fourth most common cancer globally and the leading cause of cancer-related deaths in Peru. Current synthetic treatments often fail to distinguish cancerous cells from healthy cells, resulting in severe side effects and drug resistance. This study aimed to evaluate the effects of the chloroform fraction of *Dracontium spruceanum* bulb (DSBCl) on cancer stem cells (CSCs) from AGS and KATO III gastric cancer cell lines, which represent primary and metastatic cancers. The methanolic extract was prepared and characterized via thin-layer chromatography to isolate the chloroform fraction. CSCs were identified via the CD44 marker and isolated via magnetic assisted cell sorting. The cytotoxic effect of DSBCl was evaluated to determine the IC_50_, revealing its ability to modulate CSC markers and genes associated with tumorigenesis, chemoresistance, and metastasis. In AGS CSCs, DSBCl reduced CD24 expression and downregulated the expression of genes, including *ID1, BCL2L2, ABCC2, NANOG,* and *OCT4*, while it upregulated *KLF17, BAX,* and *KLF4*. In KATO III CSCs, DSBCl increased *ID1* and *MYC* but decreased *BCL2L1* and *BAX*. These findings suggest that DSBCl modulates critical markers and genes in gastric CSCs, highlighting its potential for gastric cancer treatment.

**Supplementary Information:**

The online version contains supplementary material available at 10.1038/s41598-025-15172-9.

## Introduction

Gastric cancer ranks as the third leading cause of cancer-related mortality worldwide and is the leading cause of cancer-related death in Peru^1^. Late diagnosis is frequently associated with poor prognosis, as the primary symptoms of gastric cancer typically become apparent only in advanced stages, complicating effective treatment^2^.

The conventional drugs currently used to treat this type of cancer often cause unwanted side effects^3^. Moreover, many of these treatments fail to eliminate all cancer cells, particularly cancer stem cells (CSCs), a cell subpopulation, that are resistant to chemotherapy and radiation^4^. Cancer stem cells are responsible for cancer recurrence and metastasis^5^. CD44 is one of the most frequently used markers for identifying and isolating gastric cancer stem cells (GCSCs). It has been shown to be highly expressed in CSCs from both primary and metastatic gastric cancer cell lines^6^. CD44 positive cells display enhanced self-renewal, tumorigenicity, and chemoresistance, making this marker suitable for cancer stem cell enrichment^6–11^.

Identifying new sources of drugs that can effectively combat cancer cells is crucial. One promising alternative is the use of active compounds found in medicinal plants^12^. Numerous chemotherapy drugs, such as paclitaxel from *Taxus brevifolia* and vincristine from *Catharanthus roseus,* are derived from natural sources. These compounds have been used as chemotherapeutics and have shown the capacity to target CSCs and exhibit anticancer properties^13,14^. Several reports in the literature have highlighted the anticancer properties of medicinal plants. NAPRALERT is a primary database cataloging chemical compounds extracted from plants^15^.

Among the plant-based therapeutic candidates, *Dracontium spruceanum*, traditionally used in Amazonian medicine, has gained attention for its potential anticancer properties. Previous studies have shown that its extracts exert cytotoxic effects on breast cancer cell lines^16^. In phytochemical research, liquid–liquid solvent partitioning (eluotropic separation) is commonly applied to isolate compound classes based on polarity. The chloroform fraction *of D. spruceanum* is expected to concentrate low- to medium-polarity secondary metabolites, such as terpenoids, flavonoids, and sterols, which have been widely associated with anticancer activity^12,17^. Flavonoids such as quercetin have been shown to inhibit gastric cancer progression through apoptosis, suppression of PI3K/Akt signaling, and reduction of cancer stem cell viability^18,19^. Moreover, terpene-rich extracts from related medicinal plants have demonstrated inhibitory effects on gastric cancer cell invasion and cytoskeletal rearrangement^20^. Furthermore, preliminary screening with *D. spruceanum* extracts and fractions indicated that the chloroform fraction exhibited greater cytotoxicity than other solvent fractions when tested in gastric cancer cell lines^21^.

Numerous plant extracts and phytochemicals have demonstrated cytotoxic activity against gastric cancer. For instance, wogonin, a flavonoid from *Scutellaria baicalensis*, has been shown to inhibit proliferation, induce apoptosis and G0/G1 cell cycle arrest, and suppress migration and invasion in gastric cancer cell lines such as SGC-7901 and BGC-823^22^. Curcumin, the principal polyphenol from *Curcuma longa*, has demonstrated inhibition of proliferation, migration, and invasion in MKN-45 and SGC-7901 cells by modulating PAK1 and NF-κB signaling pathways^23^. Additionally, triptolide, a diterpenoid isolated from *Tripterygium wilfordii*, suppresses IL-1β induced IL-8 in AGS cells by inhibiting ROS/ERK/AP-1 and ROS/NF-κB pathways, attenuating tumor angiogenesis^24^.

This study aimed to evaluate the impact of the chloroform fraction of *D. spruceanum* on markers associated with tumor development, chemotherapy resistance, and the spread of gastric CSCs. These findings provide a potential alternative for future therapies targeting gastric CSCs and underscore the importance of medicinal plants.

## Results

### Chloroform fraction obtained from the methanolic extract of *Dracontium spruceanum* bulbs

**Fig. 1:**
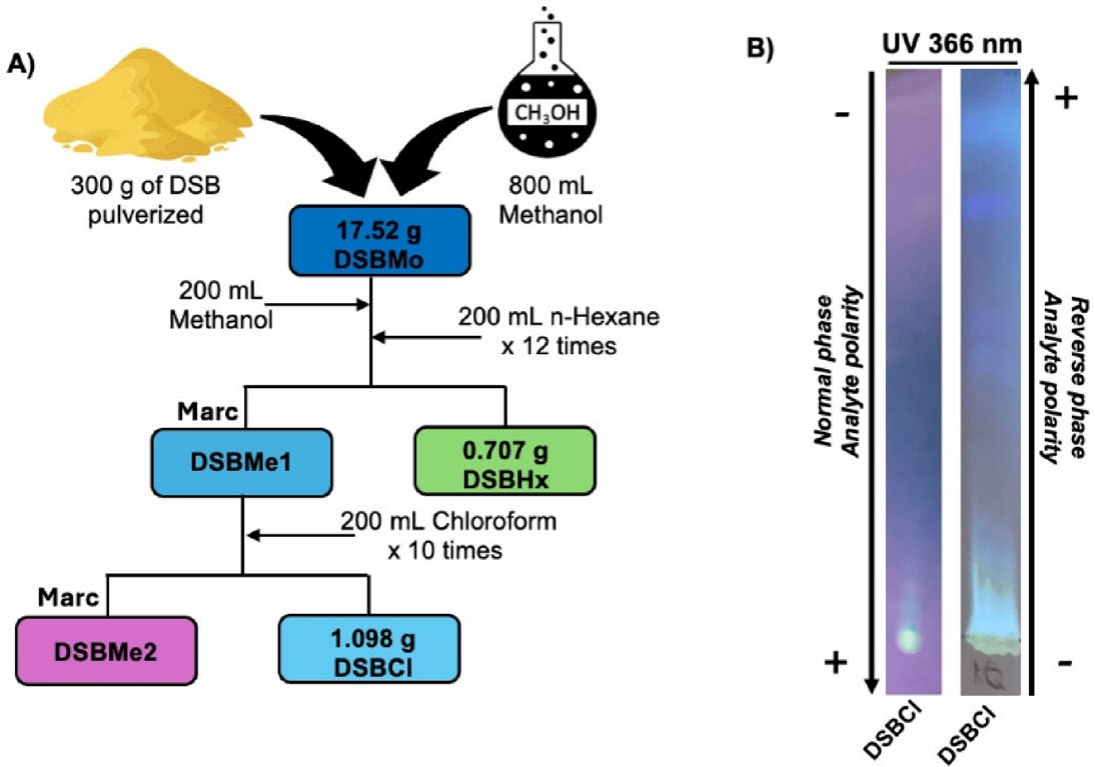
(**A**) Flow diagram showing dry weights from the liquid‒liquid fractionation of the methanolic extract of *D. spruceanum* bulbs (DSBMo). The fractions included DSBHx (hexane), DSBCl (chloroform), and DSBMe1/DSBMe2 (remnants). (**B**) Thin-layer chromatography (TLC) of DSBCl in normal and reversed-phases under longwave UV light. The normal phase shows a nonpolar compound spot at the start. The reversed-phase reveals bands of compounds with varying nonpolar characteristics. The black arrows indicate polarity. Normal phase: Silica gel 60 G F₂₅₄, benzene:acetone (15:1). Reversed-phase: Silica R8, benzene:acetone:ethyl acetate (15:1:2).

To isolate the chloroform fraction (DSBCl) from *Dracontium spruceanum* bulb, a methanolic extract (DSBMo) was first prepared, yielding a dry mass of 17.5 g. The extract was then subjected to liquid–liquid fractionation using hexane as the first solvent, resulting in a hexane fraction (DSBHx) with a dry mass of 0.707 g. Subsequently, the remaining methanolic phase (DSBMe1) was partitioned with chloroform, yielding 1.098 g of the chloroform fraction (DSBCl) (Fig. 1A). A total of 32 mg of DSBCl was resuspended in 1 mL of 100% DMSO to prepare a stock solution at a concentration of 32 mg/mL, which was aliquoted and stored at − 80 °C for further experiments.

### Thin layer chromatography (TLC) and reverse-phase TLC

Similar patterns were observed with long-wavelength UV (UV 366 nm) and short-wavelength UV (UV 254 nm) light, revealing the presence of aromatic compounds and highly conjugated complexes, which absorb UV light. To demonstrate the polar compounds present in DSBCl, reverse TLC was subsequently carried out, using a solvent mixture of benzene–acetone–ethyl acetate (15:1:2) as the mobile phase, with different bands being observed at different levels when exposed to longwave UV light (Fig. 1B).

### Isolation and characterization of AGS CSCs

**Fig. 2:**
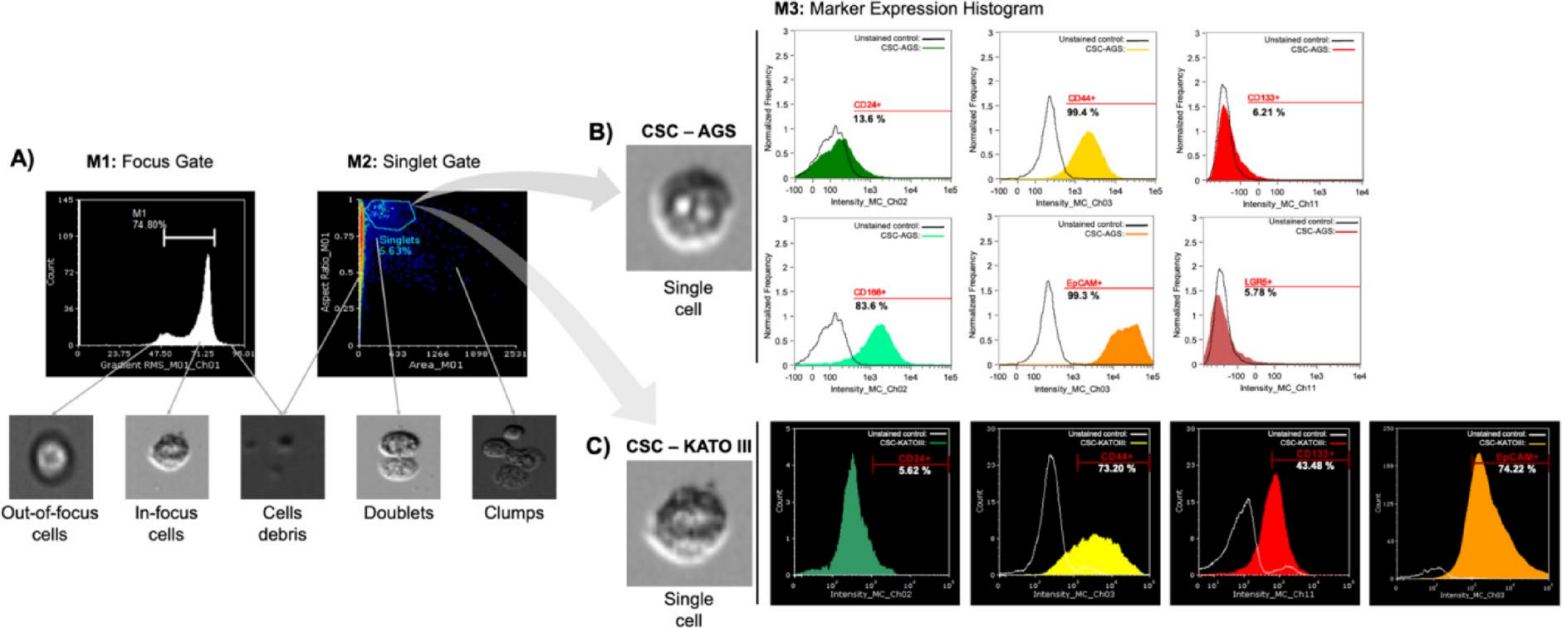
Flow cytometry characterization of gastric cancer stem cells (CSCs). (**A**) Gating strategy to exclude out-of-focus cells, debris, doublets and cell clumps. (**B**) Characterization of AGS CSCs, showing high expression of CD44+, CD166+, and EpCAM+. (**C**) Characterization of KATO III CSCs, which displayed high levels of CD44+, CD133+and EpCAM+.

To isolate and enrich cancer stem-like cells (CSCs) from the AGS gastric cancer cell line, flow cytometry was performed before and after magnetic sorting based on CD44 expression. Prior to selection, 66.79% of the AGS cells expressed CD44. Following magnetic isolation, this proportion increased to 96.33%, and 4 days later, CD44 expression reached 99.42%, confirming successful enrichment of a CD44⁺ CSC population. To further characterize the immunophenotype of the isolated cells, additional flow cytometry analysis was carried out using markers associated with gastric CSCs. The resulting profile of single cells gate (Fig. 2A) revealed expression of CD24 (13.6%), CD133 (6.21%), EpCAM (99.3%), CD166 (83.6%), and LGR5 (5.78%). These expression patterns, visualized using AMNIS flow cytometry, suggest a heterogeneous population with a core identity marked by high EpCAM and CD166 expression (Fig. 2B).

### Isolation and characterization of KATO III CSCs

To isolate and characterize CSCs from KATO III, we performed flow cytometry before and after cell sorting to confirm the isolation of CSCs. The flow cytometry results revealed that 50.08% of the cells were CD44 positive before sorting, which increased to 69.79% after magnetic selection. Importantly, due to the suspension growth pattern of KATO III cells and their propensity to form clusters, CD44^−^ cells might have remained physically associated with CD44⁺ cells despite enzymatic dissociation with Accutase. This persistent cell–cell adhesion could have resulted in the co-isolation of CD44^−^ cells during magnetic separation using anti-CD44 antibodies, which may explain the presence of 30.21% CD44^−^ cells in the sorted population (69.79% CD44⁺). Notably, after 4 days of culture, the proportion of CD44⁺ cells increased to 78.5%, suggesting a gradual reduction in CD44^−^ cells over time, likely due to the preferential survival and proliferation of the CD44⁺ subpopulation under the given culture conditions. This confirms the successful isolation and selection of CSCs with the desired immunophenotype. Additionally, we performed another flow cytometry analysis of KATO III CSCs to characterize the membrane immunophenotypes of markers related to CSCs. The results revealed that these cells expressed CD24, CD44, CD133, and EpCAM at percentages of 5.62%, 73.20%, 43.48%, and 74.22%, respectively, which was also confirmed by images taken with an AMNIS flow cytometer (Fig. 2C).

### Cytotoxic effects of solvents on AGS and KATO III cell lines

#### Dimethyl sulfoxide toxicity

**Fig. 3:**
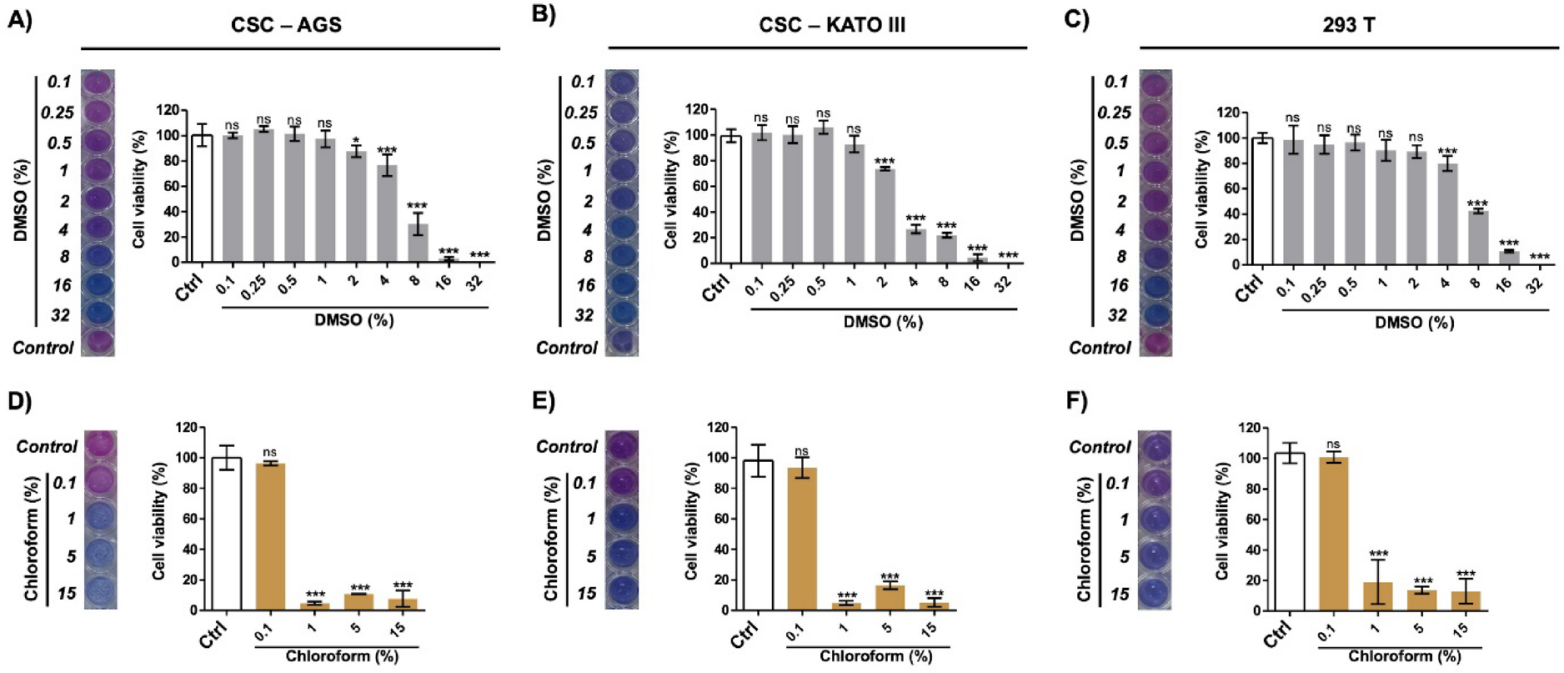
Cell viability at increasing concentrations of DMSO (0.1%, 0.25%, 0.5%, 1%, 2%, 4%, 16%, and 32%) in AGS (**A**), KATO III (**B**), and 293T (**C**) cell lines and at increasing concentrations of chloroform (0.1%, 1%, 5%, and 15%) in AGS (**D**), KATO III (**E**), and 293T (**F**) cell lines. The left side of each histogram shows the results from the resazurin assay, where viable cells reduce the reagent to a pink color, whereas nonviable cells retain the resazurin blue color. Ctrl: Cells in culture medium (control group). Statistical significance levels compared with the control group: *p < 0.05; **p < 0.01; **p < 0.001; *ns* not significant.

In this study, DMSO was the vehicle used to dissolve the dry weight of DSBCl. Therefore, before conducting cytotoxicity assays of DSBCl on CSCs, the effect of the vehicle was evaluated to avoid bias in the results. A cytotoxicity assay was performed at DMSO concentrations of 0.1%, 0.25%, 0.5%, 1%, 2%, 4%, 8%, 16%, and 32% in three cell lines: AGS, KATO III, and 293T. After 24 h of exposure, in all the cell lines at concentrations of 2% DMSO or lower, the cells maintained their morphology, adhering to the plate (AGS and 293T) or being suspended with intact and refringent membranes (KATO III). They also reduced resazurin to pink resorufin, indicating that cell viability was not significantly different from that of the control (Fig. 3A–C). In contrast, at concentrations of 4% and higher in DMSO, a toxic effect was observed in all the cell lines. As the concentration of DMSO increased, the cells showed signs of stress, beginning to contract and detach from the plate (in the case of AGS and 293T) or starting to disaggregate, resulting in small, contracted cells with cellular fragments in suspension (in the case of KATO III). Compared with those in the control wells, they no longer reduced the amount of resazurin and remained blue in the culture plate wells, indicating that the cells were dying or dead. After 48 h of incubation, a similar effect was observed, but the cytotoxic effects on AGS and KATO III cells began at a DMSO concentration of 2% (Fig. 3B).

#### Chloroform toxicity

Although the dry weight of DSBCl contained minimal traces of chloroform, as it was evaporated in the rotary evaporator, a cytotoxicity assay was performed at chloroform concentrations of 0.1%, 1%, 5%, and 15% in three cell lines: AGS, KATO III, and 293T. The assay revealed that at chloroform concentrations less than 0.1%, all the cell lines maintained viability, with no significant difference compared with the control. However, chloroform concentrations of 1% and higher were toxic to all the cell lines, resulting in a significant decrease in cell viability (*p* < 0.001) (Fig. 3D–F). Additionally, in 5% chloroform, micelle formation and phase separation were observed due to the nonpolar nature of chloroform (Supplementary Fig. 2).

### Cytotoxicity of the chloroform fraction of the methanolic extract of *Dracontium spruceanum* in gastric cancer stem cells

CSCs isolated from AGS and KATO III cell lines were exposed to concentrations of 1.25, 2.5, 5, 10, 20, 40, 80, and 160 µg/mL DSBCl. Moreover, the same assay was performed under the same conditions in the 293T cell line, which was used as a cytotoxicity control for DSBCl in noncancerous cells.

**Fig. 4:**
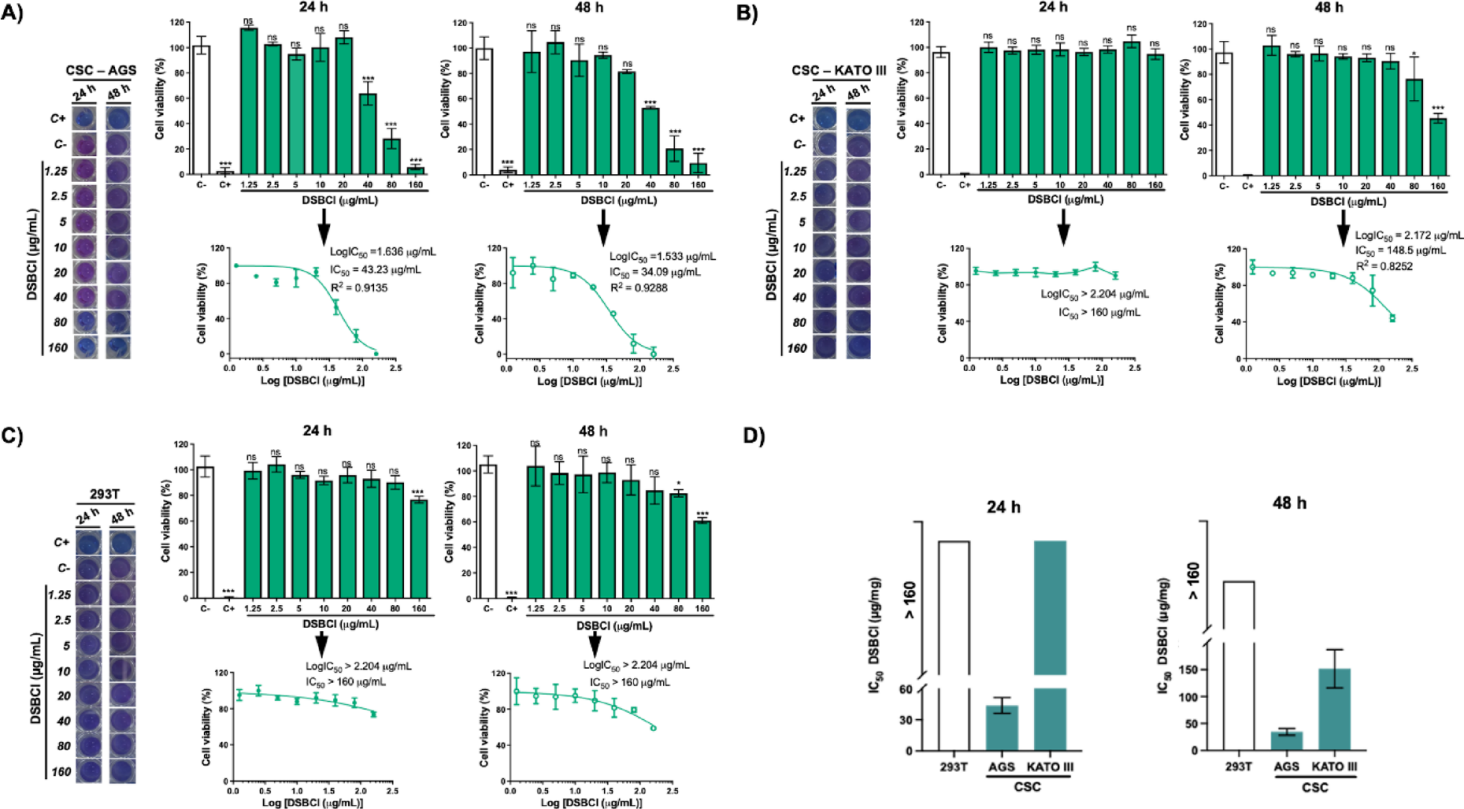
Cellular cytotoxicity of DSBCl at concentrations of 1.25, 2.5, 5, 10, 20, 40, 80, and 160 µg/mL in AGS CSCs (**A**), KATO III CSCs (**B**), and 293T cells (**C**) after 24 and 48 h of treatment. Cell viability was assessed via a resazurin assay, where live cells reduce resazurin (blue) to resorufin (pink), whereas dead cells retain blue. The bar graphs show the mean cell viability (%) ± SD, and the dose‒response curves represent the IC_50_ values. (D) Comparison of IC_50_ values for 293T, AGS, and KATO III CSCs at each time point. C+: cells treated with 15% DMSO in culture medium (positive cytotoxicity control). C−: cells treated with 0.1% DMSO in culture medium (vehicle control). Statistical significance: *p < 0.05; **p < 0. 01; ***p < 0.001; *ns* not significant.

The results of the cytotoxicity assay revealed that the viability of CSCs from AGS exposed to DSBCl for 24 h was not affected by a concentration of 20 µg/mL of DSBCl, at which time the CSCs adhered to the plate, maintaining their morphology to some extent and reducing resazurin to pink resorufin. However, above 20 µg/mL, changes in the morphology of AGS CSCs were observed, and these cells appeared stressed and detached (Supplementary Fig. 3A). As the concentration of DSBCl increased, a decreased color change in the resazurin medium (blue) was observed due to its lack of reduction. The maximum IC_50_ of DSBCl was 43.23 µg/mL. After 48 h of exposure to DSBCl, a similar effect was observed in AGS CSCs as at 24 h, but the IC_50_ was 34.09 µg/mL (Fig. 4A).

In KATO III CSCs, 24 h of exposure to DSBCl had no marked effect on their viability, with cells generally in suspension with intact and refractive membranes (Supplementary Fig. 3B), reducing resazurin to pink resorufin. Although the cells were viable, at higher concentrations of DSBCl, the cells were stressed; thus, the IC_50_ of DSBCl may be greater than 160 µg/mL (Fig. 4B). After 48 h of exposure, KATO III CSCs were affected, starting at a concentration greater than 80 µg/mL, at which time the cells began to form small dark groups of dead cells with cellular fragments in suspension, and they no longer reduced resazurin. Quantitative analysis revealed that these cells significantly differed from the control, with an IC_50_ of 148.5 µg/mL (Fig. 4B).

The results of the cytotoxicity control assay in noncancer 293T cells revealed that at 24 and 48 h of exposure to DSBCl, there was no marked effect on their viability, with 293T cells having intact cell membranes, adhering to the plate, and forming interacting cell groups (Supplementary Fig. 3C), reducing resazurin to resorufin. In this case, at higher concentrations of DSBCl, the cells were stressed; thus, the IC_50_ of DSBCl may be greater than 160 µg/mL for both 24 and 48 h (Fig. 4C).

Finally, a comparative analysis of the IC_50_ values obtained for AGS and KATO III CSCs versus the 293T cell line was performed. The results showed that at 24 h, DSBCl had a significant effect on AGS CSCs, and at 48 h, DSBCl had a greater effect on both AGS and KATO III CSCs (Fig. 4D).

### Effect of DSBCl on the expression of AGS CSC markers

**Fig. 5:**
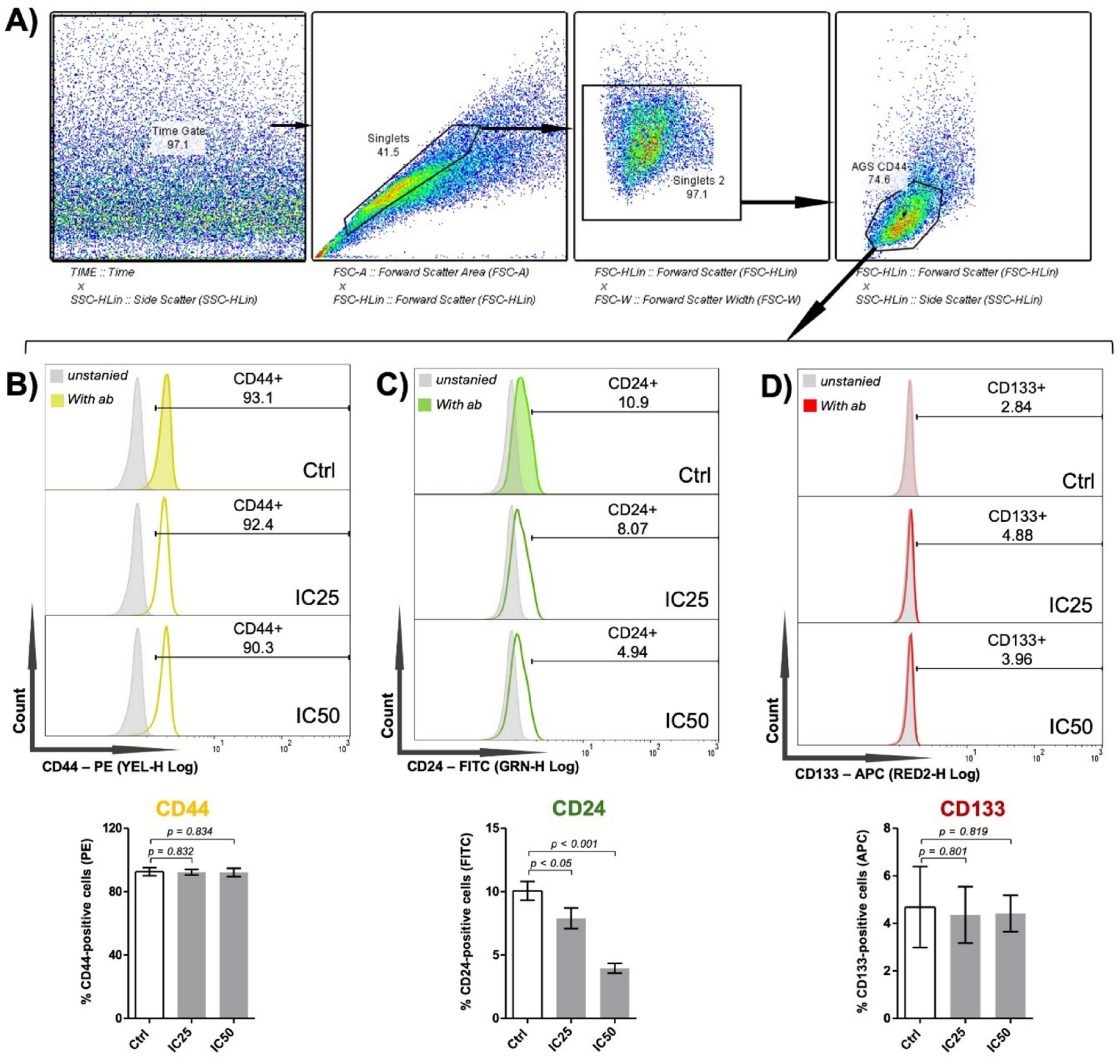
DSBCl effects on AGS CSC markers. (**A**) Gating strategy for flow cytometry analysis. Histogram and bar graph of flow cytometry data showing CD44 (**B**), CD24 (**C**), and CD133 (**D**) markers in AGS CSCs treated with the IC_25_ and IC_50_ of DSBCl. Ctrl: cells treated with 0.1% DMSO in culture medium (vehicle control).

After the IC_50_ value of DSBCl for AGS CSCs was determined, an assay was performed using the IC_25_ and IC_50_ of DSBCl to evaluate its effect on CSC markers. Flow cytometry analysis of gated single cells (Fig. 5A) showed no significant effect on the expression of CD44 (Fig. 5B) or CD133 (Fig. 5D), but a significant reduction in the expression of CD24 compared with the control (Fig. 5C).

### Gene expression analysis by qPCR

**Fig. 6:**
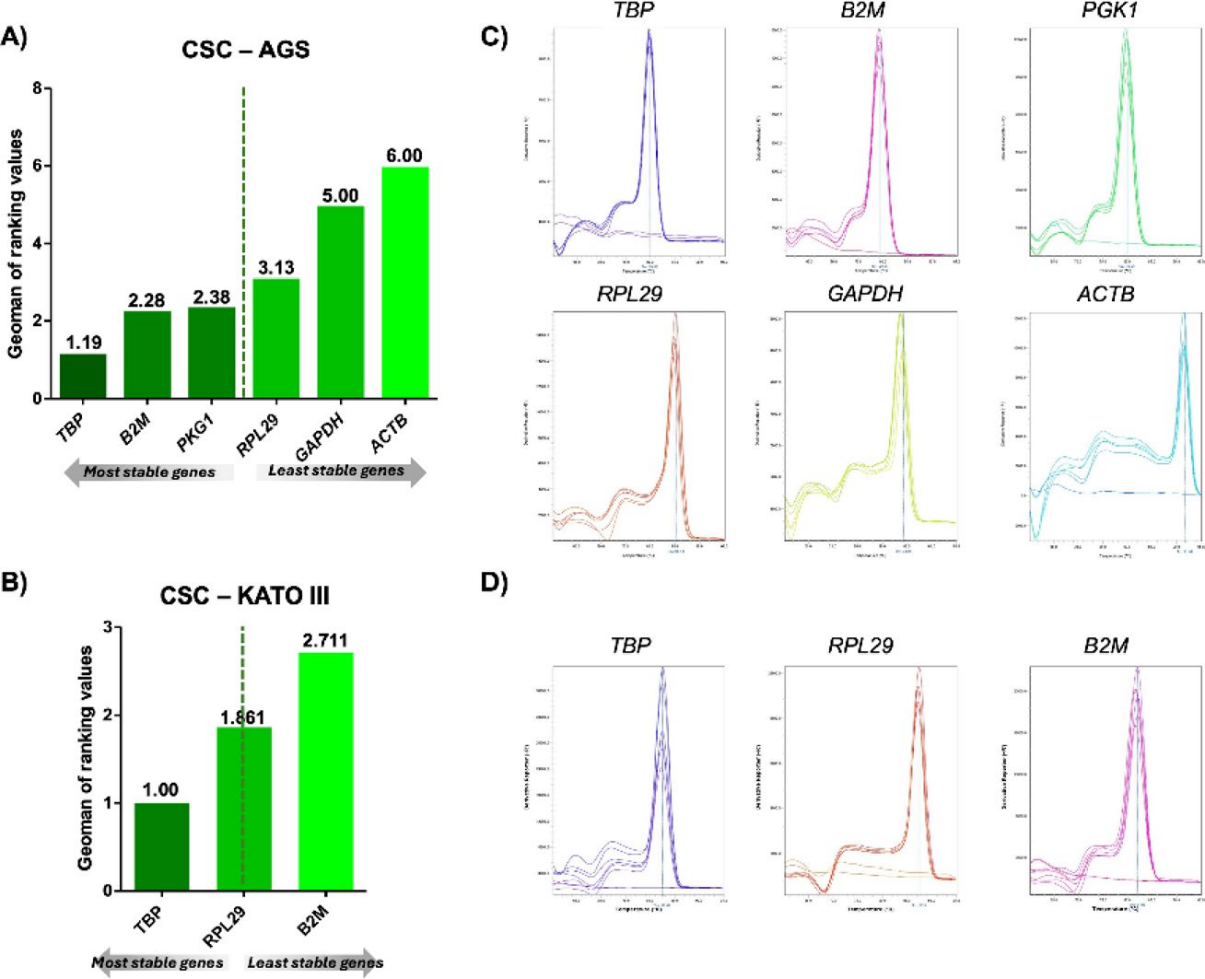
Stability of housekeeping genes and melt curve analysis in gastric CSCs. Stability ranking of housekeeping genes in AGS (**A**) and KATO III (**B**) CSCs treated with DSBCl. Melting curves for housekeeping primers in AGS (**C**) and KATO III (**D**) CSCs, showing single Tm peaks and no primer-dimer formation.

The following results were obtained for AGS CSCs, from the most stable to the least stable genes: *TBP*, *B2M*, *PKG1*, *RPL29*, *GAPDH*, and *ACTB*, with scores of 1.19, 2.28, 2.38, 3.13, 5.00, and 6.00, respectively (Fig. 6A,C). The housekeeping genes TBP and B2M are the most stable under these conditions. The results for KATO III cells, from the most stable to the least stable genes, are as follows: *TBP*, *RPL29*, and *B2M*, with scores of 1, 1.861, and 2.711, respectively (Fig. 6B,D), with *TBP* and *RPL29* being the most stable. On the basis of these findings, *TBP* was selected as the reference gene for AGS CSCs. For KATO III cells, *RPL29* was chosen as the reference gene because of the depletion of the *TBP* primer stock.

### Effects of DSBCl on the gene expression of AGS and KATO III CSCs.

**Fig. 7:**
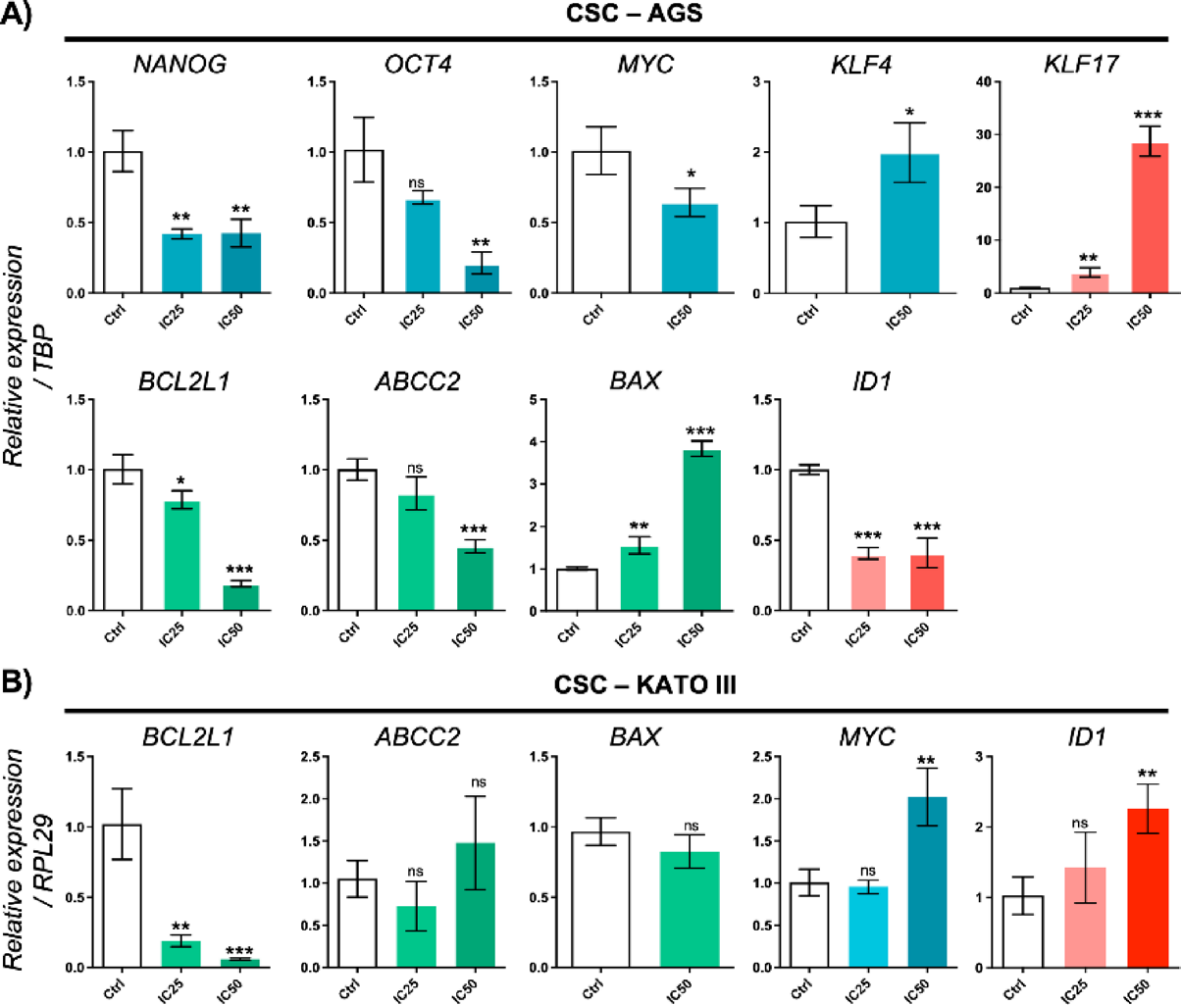
Relative gene expression analysis in gastric CSCs treated with DSBCl. qPCR analysis of AGS and KATO III CSCs treated with the IC_25_ and IC_50_ of DSBCl. The bar graphs show the fold changes in genes involved in tumorigenesis, chemoresistance, and metastasis: NANOG, OCT4, MYC, KLF4, KLF17, BCL2L1, ABCC2, BAX, and ID1 in AGS CSCs (**A**) and BCL2L1, ABCC2, BAX, MYC, and ID1 in KATO III CSCs (**B**). Gene expression was normalized to that of the TBP and RPL29 housekeeping genes. Statistical significance: *p < 0.05; **p < 0.01; ***p < 0.001; *ns* not significant.

RNA was extracted from AGS and KATO III CSCs following 24 and 48 h of exposure to DSBCl (Supplementary Fig. 4). In AGS CSCs, a reduction in the expression levels of the genes *BCL2L1*, *ABCC2*, and *OCT4* was observed in a concentration-dependent manner upon exposure to DSBCl. The expression of the *ID1* and *NANOG* genes was also reduced, but there was no significant difference between the IC25 and IC_50_ values of DSBCl. Moreover, the expression of the genes *KLF17* and *BAX* increased in a concentration-dependent manner with DSBCl, with the expression of *KLF17* increasing almost 30-fold. Finally, the expression of the genes *MYC* and *KLF4* increased when the cells were treated with the IC_25_ of DSBCl but decreased when the cells were exposed to the IC_50_ of DSBCl (Fig. 7A).

In KATO III cells, the expression of BCL2L1 was significantly reduced in a concentration-dependent manner with DSBCl. The expression of the *ID1* and *MYC* genes increased when the cells were exposed to the IC_50_ of DSBCl but not to the IC_25_ of DSBCl. Finally, there was no effect of DSBCl on *ABCC2* and *BAX* expression (Fig. 7B).

## Discussion

The aim of this study was to investigate the impact of the chloroform fraction of *D. spruceanum* (DSBCl) on biomarkers linked to tumor initiation, drug resistance, and metastatic ability in gastric cancer stem cells (GCSCs) obtained from AGS and KATO III cell lines.

The TLC analysis of DSBMo revealed traces of steroidal sapogenins, flavonoids, diterpenes, steroids, and quinones. Similar findings were reported by Sandoval et al.^25^, who identified flavonoids, steroids, alkaloids, and catechins in a related study. Additionally, research at Sao Paulo University revealed that the ethanolic extract of the bulbs or rhizome of *D. spruceanum* primarily consists of lipid metabolites such as sitosterol and estigmasterol^26^, which likely overlap with those identified in the DSBMo extract discussed in this study. These secondary metabolites have demonstrated antioxidant, anti-inflammatory, and cytotoxic effects on breast cancer cell lines^16^.

DSBCl was obtained to isolate low-polarity compounds from the methanolic extract, as suggested by TLC analysis. DSBCl predominantly contains compounds of low and medium polarity, as indicated by their retention at the origin point of the sample. Reverse-phase chromatography further confirmed the presence of low-polarity compounds, with a notable spot at the upper end and a smear from the origin point. Studies have demonstrated that chloroform fractionation effectively separates low-polarity compounds such as terpenoids, flavonoids, fats, and oils^17^.

Storage conditions, time, and temperature are factors to consider when working with extracts, as they can affect the composition or concentration of metabolites present in the extracts or fractions of medicinal plants. As shown in the chromatographic profile of DSBMo, it changed over time (Supplementary Fig. 5). This is because many compounds can be thermo or photosensitive and are susceptible to degradation due to storage time^27^, potentially leading to the loss of the active compounds of interest. Studies conducted with blueberry extracts stored at different temperatures (− 20, 6, 23, and 35 °C) revealed that both the composition of metabolites and the cytotoxic activity were affected by storage at temperatures above 6 °C^28^.

Studies have shown that cells from primary tumors and gastric cancer cell lines isolated via the CD44 marker exhibit characteristics of CSCs. Specifically, CD44+ cells have demonstrated tumorigenic capacity both in vivo and in vitro, unlike CD44− cells, which lack this capacity^6–8^. This study further supports these findings by demonstrating the tumorigenic capacity of GCSCs isolated with the CD44 marker in vitro.

Additionally, as part of CSC characterization, analysis of the cell membrane surface immunophenotypes via flow cytometry is mandatory. CSCs isolated from AGS and KATO III cells presented different percentages of expression of these markers. AGS CSCs (AGS CD44+) expressed more EpCAM and CD166 (> 80%) and less CD24, CD133, and LGR5 (< 14%). This result is consistent with the CD24- and CD44-positive cells identified in AGS CSCs via the same markers^29^, as well as the low expression of CD133^9,10^. KATO III CSCs (KATO III CD44+ cells) expressed more EpCAM (> 70%), intermediate levels of CD133 (> 70%), and less CD24 (< 10%). These results are consistent with those reported in a study from Korea, in which KATO III CSCs expressed more CD133 and less CD24 and CD44^9,11^.

The difference in the expression patterns of membrane immunophenotypes between AGS and KATO III CSCs is likely due to their cellular characteristics. AGS CSCs are adherent cells from primary gastric cancer, whereas KATO III CSCs grow in suspension and are derived from metastatic cancer. Additionally, the expression of CD133 differed among the groups. This protein is related to maintaining CSC characteristics and migration through interactions between cells and the extracellular matrix and is stimulated by hypoxia in the tumor microenvironment through the regulation of HIF-1α^30^. These characteristics are more common in metastatic cancer cells than in primary cancer cells.

With respect to the cytotoxic activity of DSBCl on GCSCs, several studies have identified chemical compounds with cytotoxic and anticancer activity present in *D. spruceanum*. For example, methanolic extracts prepared from the leaves and bulbs of *D. spruceanum* have shown activity against breast cancer cells^16^. In this study, DSBCl evidenced cytotoxicity against AGS CSCs with IC_50_ values of 43.23 and 34.09 µg/mL at 24 and 48 h, respectively. In contrast, KATO III CSCs were less sensitive with IC_50_ value greater than 160 µg/mL at 24 h and 148.5 µg/mL at 48 h. This disparity in sensitivity is likely due to the intrinsic chemoresistance of KATO III CSCs, which originate from metastatic gastric tumors^31^. Previous studies have shown that KATO III cells exhibit significantly greater resistance to platinum-based chemotherapeutic agents compared to AGS cells, which are derived from a primary tumor. For instance, KATO III cells have shown approximately 2.5-fold higher resistance to cisplatin (IC_50_: 35.7 µM vs. 14.6 µM in AGS) and 2.6-fold higher resistance to oxaliplatin (IC_50_: 37 µM vs. 9.9 µM in AGS)^32^. These findings are consistent with our results and reinforce the concept that CSCs from metastatic origins, such as KATO III, exhibit heightened resistance to cytotoxic agents, including those derived from natural products.

Importantly, the IC_50_ values observed for both AGS and KATO III CSCs were significantly lower than those reported for 293T noncancerous cells, indicating a preferential cytotoxic effect of DSBCl on GCSCs. This selectivity suggests potential therapeutic relevance and warrants further investigation.

Comparable cytotoxic activities have been reported for other plant-derived extracts tested on gastric cancer cell lines. For example, the ethyl acetate extract of *Elephantopus mollis* exhibited an IC_50_ of 27.5 µg/mL in AGS cells^33^, while the chloroform fraction of *Piper aduncum* showed IC_50_ values of 49.47 µg/mL in AGS and 64.68 µg/mL in KATO III cells after 48 h of treatment^34^. These values are within the same range as those observed for DSBCl, supporting its potential as an anticancer agent.

Although direct comparisons between DSBCl and standard chemotherapeutic drugs were not performed in this study, previously reported IC₅₀ values for 5-fluorouracil (5-FU) in gastric cancer cells provide useful benchmarks. For instance, 5-FU has been shown to have IC_50_ values of approximately 60 µg/mL in AGS cells and 40 µg/mL in BGC-823 cells after 48 h of exposure^35^. Considering that CSCs are generally more resistant than parental tumor cells, the cytotoxic activity of DSBCl observed in our study underscores its potential as a candidate for further development in gastric cancer therapy.

While this is the first study to demonstrate the effect of DSBCl on GCSCs, it has been reported that DSBCl has an IC_50_ in AGS cells of 82.52 and 25.35 µg/mL for 24 and 48 h, respectively^21^, demonstrating that DSBCl exhibits cytotoxic activity in both AGSs and GCSCs. The possible contributors to this cytotoxic activity of DSBCl in GCSCs are terpenoids, flavonoids, fats, and oils^15^. Several bioactive compounds known to have anticancer properties are terpenoids or flavonoids.

The present study revealed a significant reduction in the expression of CD24 on the membrane surface of AGS CSCs treated with DSBCl compared with that in untreated cells. The membrane expression of CD24 has been reported to be associated with increased invasiveness, migration, and lymph node metastasis in gastric cancer^36,37^. Functionally, CD24 functions as a ligand for P-selectin, which is present on the surfaces of activated platelets and endothelial cells^38^. Moreover, CD24 expression is induced under hypoxic conditions^39^, contributing to tumor progression^40^. Although CD24 expression is linked to aggressive tumor behaviour, in vitro gastric CSCs typically exhibit a CD44-positive/CD24-low phenotype. This may be explained, at least in part, by the absence of hypoxic stimuli in standard culture conditions, which limits CD24 induction. Therefore, the observed downregulation of CD24 following DSBCl treatment may reflect the suppression of pro-metastatic and migratory pathways, supporting the potential anti-invasive properties of this plant-derived fraction.

CSCs are known to exhibit chemoresistance, primarily due to the expression of transporter proteins associated with multidrug resistance, including *ABCC2* (*MRP2*). This protein is involved in the ATP-dependent transport of lipophilic substance conjugates, functioning as an export pump^41,42^. Additionally, BCL-2 family proteins play crucial roles in cellular apoptosis, with members exhibiting either proapoptotic or antiapoptotic functions^43^. In cancer cells, antiapoptotic BCL-2 family proteins are often overexpressed, thereby inducing resistance to cell death caused by chemotherapeutics and radiotherapy^44^. Among these antiapoptotic proteins is BCL2L1, also known as BCL-X, which regulates its antiapoptotic function through its BCL-X_L isoform by forming heterodimers with the proapoptotic proteins BAK and BAX^45^. In the present study, DSBCl significantly reduced the expression of *ABCC2* and *BCL2L1* (BCL-X_L isoform) in AGS CSCs and KATO III metastatic cells while concurrently increasing the expression of *BAX* in AGS CSCs. These findings indicate a general inhibition of chemoresistance in these cells. Furthermore, certain terpenoids, such as d-limonene and triptolide^12,46^, and flavonoids^46^, such as quercetin and naringenin^47,48^, which are isolated from medicinal plants, can inhibit the expression of *ABCC2* and *BCL-X_L* in cancer cells.

The study also revealed that DSBCl significantly reduced the expression of genes related to tumorigenesis and pluripotency in GCSCs, including *NANOG*, *OCT4*, and *cMYC*. These genes are key regulatory factors for maintaining undifferentiated stem cells^49^, are significantly overexpressed during the progression of gastric cancer, and can serve as potential prognostic biomarkers for this type of cancer^50^. Therefore, DSBCl has potential as an inhibitor of GCSC progression. DSBCl also induced the overexpression of *KLF4* (*p* < 0.05) and *KLF17* (*p* < 0.001) in AGS CSCs. KLF family genes play important roles in both normal development and carcinogenesis. Among these factors, *KLF4* is particularly associated with the regulation of proliferation, differentiation, and tumorigenesis of the gastrointestinal tract epithelium. *KLF4* represses several cell cycle regulatory gene promoters, such as cyclin D1 and ornithine decarboxylase, and induces cell cycle arrest at the G1/S boundary, making *KLF4* a negative regulator of cell growth^51^. Additionally, some studies suggest that *KLF4* may have a suppressive effect on proliferation and metastasis^52^. *KLF17* is poorly expressed in GCSCs, and its overexpression reportedly inhibits epithelial‒mesenchymal transition in gastric cancer cells through the TGF-β/Smad pathway, thereby reducing the invasion and migration of these cells^53,54^. Thus, DSBCl could inhibit the cell cycle and prevent the invasion and migration of GCSCs.

Finally, the expression of *ID1* was affected by DSBCl treatment. Specifically, *ID1* expression was significantly reduced in AGS CSCs treated with DSBCl (*p* < 0.001). Conversely, in KATO III metastatic cells treated with DSBCl, *ID1* expression was slightly induced (*p* < 0.05). This contrasting regulation suggests that DSBCl may act through distinct signaling pathways in primary versus metastatic CSCs, as multiple upstream regulators can modulate *ID1* transcription^55^. *ID1* is a known regulator of proliferation, stemness, and survival in various cancer types^56^. Its downregulation in AGS CSCs may therefore indicate inhibition of tumor-initiating capacity, aligning with an anti-stemness effect of DSBCl. In contrast, the upregulation observed in KATO III CSCs may reflect a context-dependent response, potentially related to the metastatic origin of these cells.

Moreover, *ID1* undergoes alternative splicing to generate two isoforms, Id1a and Id1b, which exert opposing effects: Id1a promotes proliferation, while Id1b suppresses proliferation and enhances self-renewal^57^. Although the present study did not distinguish between these isoforms, it is plausible that DSBCl induces differential isoform expression in AGS versus KATO III CSCs. This mechanistic complexity could contribute to the divergent regulation of *ID1* observed between the two cell lines. Additionally, previous studies have shown that *ID1* downregulation inhibits gastric cancer cell proliferation, whereas its upregulation alone does not necessarily enhance proliferation^58^, supporting the notion that DSBCl effects on *ID1* may not be uniformly pro-tumorigenic and may depend on cellular context.

Some limitations of this study are that the chemical composition of DSBCl and its active compounds were not fully characterized, which limits our understanding of their effects. Additionally, the study was conducted in vitro; thus, the results may not be directly applicable to in vivo systems because of differences in the tumor microenvironment and the processing of compounds in living organisms. Variability in storage conditions and potential degradation of active compounds over time may also impact the consistency of the results. Moreover, as the study focused on AGS and KATO III cell lines, the applicability of these findings to other gastric cancer cell lines or patient samples has yet to be validated. Further research involving in vivo models and a broader range of gastric cancer cell lines is necessary to confirm these initial findings and explore the potential therapeutic applications of DSBCl.

This study demonstrated that DSBCl has significant antitumorigenic effects on GCSCs derived from the AGS and KATO III cell lines. DSBCl effectively reduced the expression of key biomarkers associated with tumor initiation, drug resistance, and metastatic potential, including CD24, *ABCC2*, and *BCL2L1*. Additionally, DSBCl downregulated genes related to tumorigenesis and pluripotency (*NANOG*, *OCT4*, and *MYC*) and induced the overexpression of the tumor suppressor genes *KLF4* and *KLF17*, suggesting its potential to inhibit cell cycle progression and epithelial‒mesenchymal transition. The differential effect of DSBCl on *ID1* expression between AGS and KATO III cells indicates the involvement of distinct signaling pathways. Altogether, these findings support the potential of DSBCl as a promising therapeutic candidate for targeting gastric CSCs. Future in vivo studies and chemical characterization of the active components will be essential to fully validate its translational applicability.

## Methods

### Plant material collection and identification

*Dracontium spruceanum* was collected in the city of Lamas, located in the Department of San Martin, Peru. The collection took place at altitudes ranging from 310 to 920 m above sea level, at coordinates 6° 23′ 01.8″ S, 76° 30′ 21.6″ W. This species is a shrub that typically grows to an average height of 2 m, characterized by palmate leaves and bulb-like rhizomes.

Permission for plant material collection was granted by the Peruvian National Forest and Wildlife Service (Servicio Nacional Forestal y de Fauna Silvestre, SERFOR) under Directoral Resolution No. 013-2020-MINAGRI-SERFOR. The plant material was identified by Biologist José Campos de la Cruz, as confirmed by certificate No. 190-USM-2018 issued by the San Marcos Herbarium (USM) of the Museum of Natural History, National University of San Marcos, Lima, Peru. The specimen was classified as *Dracontium* aff. *spruceanum* (Schott) G.H. Zhu.

### Preparation of the methanolic extract and chloroform fraction of *D. spruceanum*

*Dracontium spruceanum* bulbs (DSB) were initially washed and dried at 40 °C for 4 days at the Natural Products Laboratory of the Universidad Científica del Sur. Subsequently, 300 g of the finely sifted powder was combined with 800 mL of 99.9% methanol (p.a., Merck) and subjected to ultrasonication for 2 h daily for 5 days. The resulting mixture was filtered through Whatman No. 1 paper (Merck) followed by 0.45 µm syringe filters, and the filtrate was concentrated via a rotary evaporator under reduced pressure. This process was iterated five times, utilizing the remaining methanolic extract each time. The resulting methanolic extracts of DSB (DSBMo) were then incubated at 30 °C until they reached a dry weight, after which the dry mass was stored and labeled in jars.

For fractionation, 200 mL of 99.9% methanol was added to the methanolic extract, and liquid‒liquid solvent extraction was carried out. Specifically, 200 mL of hexane was added to partition the extract into polar and nonpolar phases, which were separated via decantation. This step was repeated 12 times to isolate nonpolar compounds, yielding the hexane fraction (DSBHx) of the DSBMo. Additionally, a residual portion of DSBMo (DSBM1) was subjected to further fractionation with 200 mL of chloroform, which was repeated 10 times to obtain the chloroform fraction of DSBM1 (DSBCl), resulting in 32 mg of DSBCl dissolved in 1 mL of 99.9% dimethyl sulfoxide (DMSO). The solution was stored at − 80 °C for short-term use or at − 196 °C in liquid nitrogen for long-term storage.

### Thin layer chromatography (TLC) analysis

Thin layer chromatography (TLC) was performed to analyze the presence of chemical compounds in the different solutions obtained during the extraction steps of the DSB until the chloroform fraction DSBCl was obtained. The chromatographic pattern in each step of the fractionation was evaluated via TLC plates coated with silica gel 60 G F_254_ (Merck) as the stationary phase. These plates were placed in a glass cuvette containing a mobile phase consisting of the low-polarity solvent benzene:acetone (Be:Ac) at a ratio of 1:5. In some cases, other solvents were added to achieve better resolution of the chromatograms. Finally, the developers zinc chloride, aluminum chloride, antimony III chloride, potassium hydroxide and 3,5-dinitrobenzoic acid were used, following the procedure described by Lock^59^. The developed plates were then observed under visible light and ultraviolet (UV) light at 254 nm (short wave) and 366 nm (long wave).

### Cell lines and culture

AGS (ECACC 89090402) and KATO III (ECACC 86093004) cell lines were obtained from the European Collection of Authenticated Cell Cultures (ECACC), and 293T cells (CRL-3216) were acquired from the American Type Culture Collection (ATCC). AGS and 293T cells were cultured in Dulbecco’s modified Eagle’s medium (DMEM) with F12 Ham’s nutrients (DMEM-F12) (Sigma‒Aldrich) supplemented with 10% fetal bovine serum supplemented with 1× antibiotic–antimycotic mixture (Biowest). KATO III cells were cultured with Roswell Park Memorial Institute (RPMI) 1640 medium supplemented with 20% fetal bovine serum and 1× antibiotic–antimycotic mixture. All the cells were incubated at 37 °C and 5% CO_2_ until they reached 80% confluence.

### Isolation of cancer stem cells by magnetic assisted cell sorting (MACS)

For CSC isolation, MACS was used on the basis of the enrichment of CD44+ AGS and CD44+ KATO III cells according to the manufacturer’s protocol. The cell culture medium was removed from the plates containing AGS cells, which were then washed with 2 mL of 1× phosphate-buffered saline (PBS). After the PBS was discarded, 2 mL of 1× accutase was added, and the mixture was incubated at 37 °C for 5–7 min. Once the cells were detached, 2 mL of complete culture medium was added to inhibit the activity of accutase. KATO III cells were directly harvested by culture medium centrifugation at 250×*g* for 5 min since they are cells that grow in suspension. Cell counting was carried out in a Neubauer chamber. Ten million cells from each cell line were collected in a 1.5 mL tube and centrifuged at 300×*g* for 8 min; the supernatant was discarded. The cell pellet was resuspended in 80 μL of staining buffer (Miltenyi Biotec), 10 μL of FcR blocking reagent (Miltenyi Biotec), and 10 μL of anti-CD44 magnetic microbeads (Miltenyi Biotec) and incubated in the dark at 4 °C for 15 min. Following incubation, the cells were washed with 1× PBS, centrifuged at 300×*g* for 10 min and then resuspended in 500 μL of staining buffer. Then, the cells were applied onto an MS column (Miltenyi Biotec), which was previously washed three times with 500 μL of staining buffer and attached to the MiniMACS separator (Miltenyi Biotec) to retain the cells. Both AGS and KATO III cells were labeled with anti-CD44 magnetic beads. Three consecutive washes were performed with 500 μL of staining buffer to remove any unlabeled cells. Finally, after the CD44+ cells were attached to the MS column, they were eluted by removing the column from the platform and adding 1 mL of staining buffer. A portion of the positively selected cells was cultured for 4 days, while another portion was analyzed by flow cytometry to verify the selection process.

### Flow cytometry characterization of CSCs

A total of 3 × 10^5^ AGS cells were seeded in five 100 × 15 mm tissue culture-treated plates with complete DMEM and incubated at 37 °C and 5% CO_2_ for 48 h. After incubation, the culture medium was removed, and cells were washed with 3 mL of 1× PBS. Cells were then detached by incubation with 1× Accutase for 5 min at 37 °C, followed by the addition of 2 mL of complete DMEM to neutralize the enzyme. The cell suspension was transferred to 15 mL conical tubes and centrifuged at 300×*g* for 5 min. The resulting pellet was resuspended in 1 mL of complete DMEM. A 30 µL aliquot was mixed with an equal volume of trypan blue, and viable cells were counted using a Neubauer chamber.

For KATO III cells, 7 × 10^5^ cells were seeded in two T75 flasks with complete RPMI medium and cultured under standard conditions. As KATO III is a suspension gastric cancer cell line, cells remained non-adherent and formed floating clusters. After 48 h, the culture medium containing the cell population was directly collected into 50 mL Falcon tubes and centrifuged at 300×*g* for 5 min to obtain the cell pellet. The pellet was resuspended in 1 mL of complete RPMI 1640, and viability was assessed using the trypan blue exclusion method as described for AGS cells.

A total of 1 × 10^6^ cells, from each cell line, were distributed into each 1.5 mL tube for each marker studied—CD44, CD133, CD166, CD24, LGR5, and EpCAM. When more than one marker was studied, additional tubes with cells were prepared to compensate for the signals. In all cases, propidium iodide was used as an indicator of cell viability. The 1.5 mL tubes were centrifuged at 300×*g* for 10 min. After centrifugation, the supernatant was removed, and 90–99 μL of staining buffer plus 1–10 μL of the monoclonal antibodies—anti-CD44, anti-CD133, anti-CD166, anti-CD24, anti-LGR5, and anti-EpCAM—conjugated to a fluorochrome (PE, PEvio770, FITC, Viobright FITC, APC, and PerCP Cy5.5, respectively) were added. The samples were incubated for 30 min at 4 °C in the dark. The samples were then washed with 1 mL of 1× PBS and centrifuged again at 300×*g* for 10 min, and the resulting pellet was resuspended in 200 μL of staining buffer for flow cytometry analysis.

Two different flow cytometers were used depending on the number of markers analyzed. The Guava EasyCyte flow cytometer (Merck) at Universidad Científica del Sur was used for single- or dual-marker experiments, including verification of CSC enrichment before and after MACS isolation. For multiparametric analysis of CSC markers (CD44, CD24, CD133, EpCAM, CD166, LGR5), we used the Amnis FlowSight imaging flow cytometer (Luminex) at Universidad Nacional Mayor de San Marcos, which enabled simultaneous fluorescence detection and morphological imaging. The flow cytometry data were analyzed via Guavasoft 3.4, IDEAS version 6.0, or FlowJo™ v10.8 software.

### Effect of solvents on AGS and KATO III cell viability

To evaluate the effects of solvents on gastric cancer cell lines, 5 × 10^3^ AGS, 10 × 10^3^ KATO III and 5 × 10^3^ 293T cells were seeded on 96-well plates for 24 h at 37 °C and 5% CO_2_. The number of cells used for each experiment was previously standardized (Supplementary Fig. 1). Then, the culture medium was replaced with fresh medium containing different concentrations of DMSO (0.1, 0.25, 0.5, 1, 2, 4, 8, 16 and 32%) and chloroform (0.1, 1, 5 and 15%). The plates were incubated for 24 and 48 h, after which the cell viability assay was performed with resazurin reagent, which measures mitochondrial activity through the reduction of resazurin (blue) to resorufin (pink). Twenty microliters of stock solution (0.15 mg/mL) was added to each well and incubated for 3 h. The plates were subsequently read at 570 and 600 nm wavelengths via a microplate reader (Synergy LX, Biotek).

### Effect of DSBCl on AGS and KATO III stem cell viability

Similarly, to evaluate the effect of DSBCl on gastric cancer stem cells, cytotoxicity assays were carried out. These assays consisted of the distribution and culture of 5000, 10,000 and 5000 AGS, KATO-III and 293T cells, respectively, as noncancer control, in 96-well plates for 24 h. The culture medium was then replaced with medium containing 1.25, 2.5, 5, 10, 20, 40, 80 or 160 µg/mL DSBCl for 24 or 48 h of exposure. DMSO was used as a vehicle control at a final concentration of 0.1% in all viability assays. Next, 20 µL of 0.15 µg/mL resazurin was added to each well and allowed to incubate for 3 h. After this time, the reading was carried out in a Synergy LX microplate reader (BioTek) at wavelengths of 570 and 600 nm. The results were exported in .xls format for analysis.

### Selection of the reference gene for gene expression analysis

To evaluate the molecular effects of DSBCl compounds on AGS and KATO III CSCs, gene expression studies were conducted. It was crucial to first define a reference (housekeeping) gene with constant expression upon exposure to DSBCl. Therefore, six housekeeping genes were evaluated for AGS CSCs, *ACTB*, *B2M*, *GAPDH*, *PGK1*, *TBP*, and *RPL29*, and three housekeeping genes were evaluated for KATO III cells, *B2M*, *TBP*, and *RPL29*, in both treated and untreated samples exposed to the IC_25_ and IC_50_ of DSBCl. The primers used, described in Supplementary Table 1, were obtained from the RTprimerDB (http://www.rtprimerdb.org) and PrimerBank (https://pga.mgh.harvard.edu/primerbank/) databases and were selected after bioinformatics analysis via BLAST (https://blast.ncbi.nlm.nih.gov/Blast.cgi) and the OligoAnalyzer Tool (IDT, https://www.idtdna.com/pages/tools/oligoanalyzer?returnurl=%2Fcalc%2Fanalyzer).

For the evaluation of each gene, quantitative polymerase chain reaction (qPCR) was performed in a 0.1 mL tube as follows: 10 µL of BlasTaq™ 2× qPCR MasterMix (abm), 1 µL of 100 ng/µL cDNA, 0.6 µL of 10 µM forward primer, 0.6 µL of 10 µM reverse primer, and 7.8 µL of ultrapure H_2_O. A blank was also prepared in the same way but without cDNA, using the same primers and with 1 µL of nuclease-free water. The qPCR was run in a StepOne™ Real-Time PCR System thermocycler (Applied Biosystems™) under the following conditions: 95 °C for 3 min, 40 cycles consisting of two steps: denaturation at 95 °C for 15 s and hybridization/extension at 60 °C for 1 min, followed by a melting curve.

The data obtained were analyzed with the RefFinder program, which integrates the results of the four programs most commonly used for gene expression analysis—geNorm, NormFinder, BestKeeper, and the comparative ΔΔCt method—to identify the best housekeeping gene that maintains the most stable expression when exposed to DSBCl.

### Effect of DSBCl on AGS and KATO III CSC gene expression

For this assay, AGS and KATO III CSCs (400,000 and 800,000 cells, respectively) were cultured in 6 mL of complete DMEM and RPMI 1640 in 100 mm plates, with three groups: the control, IC_25_, and IC_50_ groups. After 24 h of exposure, the culture medium was removed, the plates were gently washed with 1× PBS, and 2 mL of 1× trypsin was added and incubated for 5 min at 37 °C. After this time, the cells were collected and centrifuged at 300×*g* for 5 min. The resulting pellet was used for RNA extraction in each group via an innuPrep DNA/RNA extraction kit (Analytik Jena) following the manufacturer’s instructions. The eluted DNA and RNA were quantified at 230, 260, and 280 nm to evaluate purity and quantity via a Multi-Mode Reader (BioTek). For each sample, the A260/A280 and A260/A230 ratios were considered. Total RNA integrity was assessed by electrophoresis on 1% agarose gels. The presence of clear 28S, 18S, and 5S rRNA bands was used as quality control prior to downstream gene expression analyses. Due to technical limitations, multiple experimental samples were loaded into separate wells on the same gel, or reloaded to confirm RNA quality. Images presented in Supplementary Fig. 4 were assembled from different gel segments to provide a representative and clear visualization of all conditions. The RNA was converted to cDNA via the OneScript® Plus cDNA Synthesis Kit (abm) following the manufacturer’s instructions.

For the evaluation of each gene, a 0.1 mL tube was prepared as follows: 10 µL of BlasTaq™ 2× qPCR MasterMix (abm), 1 µL of 100 ng/µL cDNA, 0.6 µL of 10 µM forward primers, 0.6 µL of 10 µM reverse primers and 7.8 µL of ultrapure H_2_O. A blank was also prepared in the same way as in the previous case but without cDNA, using the same primers and with 1 more µL of ultrapure H_2_O. Then, qPCR was performed in a StepOne™ Real-Time PCR System thermocycler (Applied Biosystems™) under the following conditions: 1 cycle of enzyme activation at 95 °C for 3 min; 40 cycles consisting of two steps, one of denaturation at 95 °C for 15 s and another hybridization/extension at 60 °C for 1 min; and a melting curve. Finally, the relative expression of the genes studied via real-time PCR was analyzed via the double delta Ct method^60^.

### Data analysis

All the assays were performed with three technical replicates and three biological replicates. Once the data were obtained, statistical analysis was carried out via GraphPad Prism version 9.0.0 for Windows (GraphPad Software, San Diego, California, USA). For the cytotoxicity tests between the control group and the treated groups, one-way ANOVA with Dunnett’s post hoc test was performed. To calculate the IC_50_, a nonlinear regression of the normalized data was conducted. To evaluate significant differences in gene expression between groups, Student’s t test was used.

## Supplementary Information

Below is the link to the electronic supplementary material.

Supplementary Material 1

## Data Availability

The datasets generated during and/or analysed during the current study are available from the corresponding author on reasonable request.
