# Supplementary material for: The chloroform fraction of Dracontium spruceanum modulates gene expression in gastric cancer stem cells
Source: Sci Rep. 2025 Aug 27;15:31645. doi: 10.1038/s41598-025-15172-9 (PMC12391353; doi:10.1038/s41598-025-15172-9)
Supplement: Supplementary file 1 — Supplementary Material 1 [file 41598_2025_15172_MOESM1_ESM.docx]

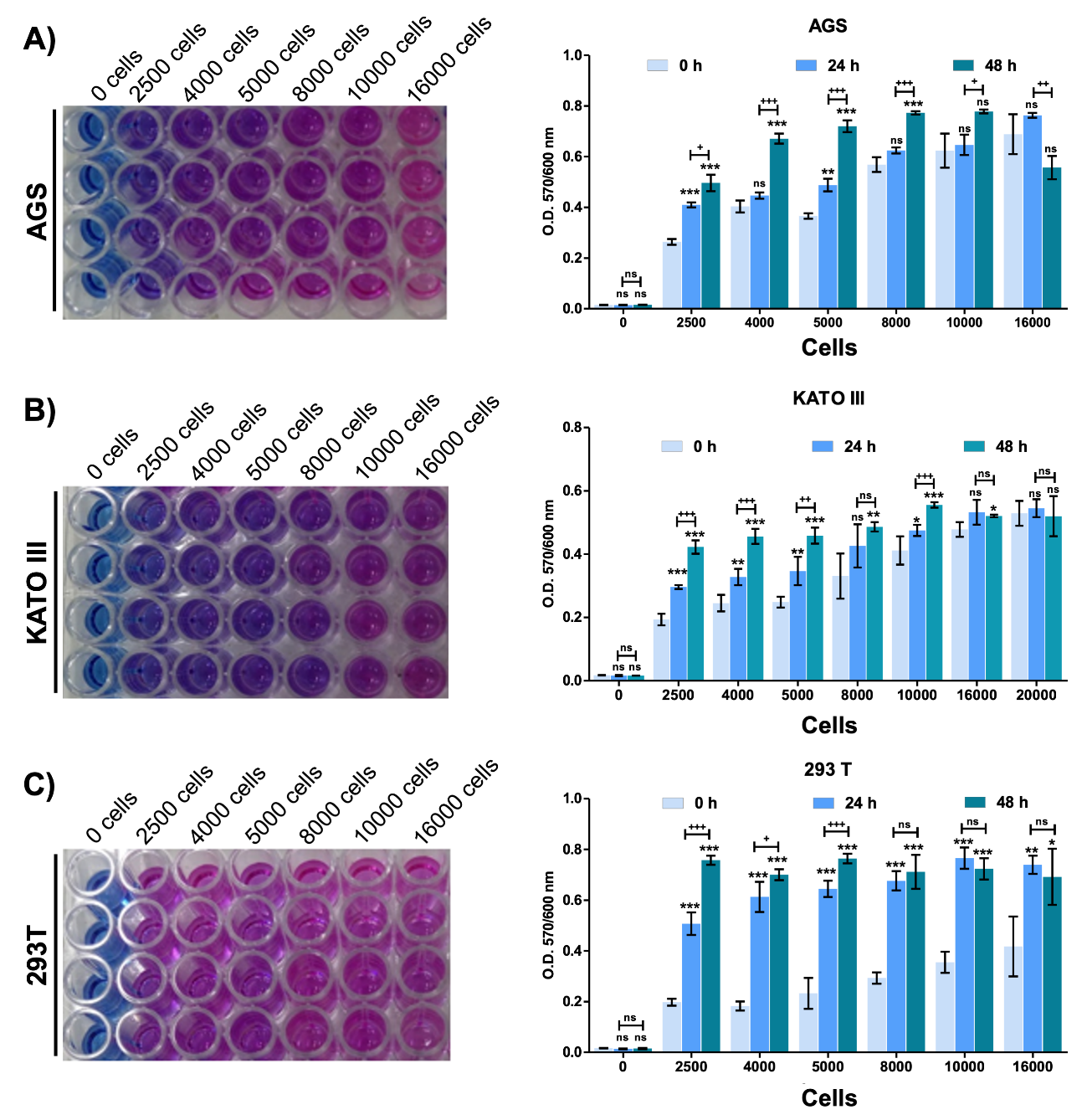


Supplementary Fig.1. Determination of optimal seeding density for Resazurin assay. AGS (A), KATO III (B), and 293T (C) cells were seeded at increasing densities (0 to 16,000 cells/well) in 96-well plates. Representative images of crystal violet staining (left) and corresponding quantification by absorbance at 570/600 nm (right) are shown at 0 h, 24 h, and 48 h. Data represent mean ± SEM from three independent experiments. Statistical analysis was performed using one-way ANOVA with Tukey’s multiple comparison test (p < 0.05, *p < 0.01, **p < 0.001; ns, not significant).


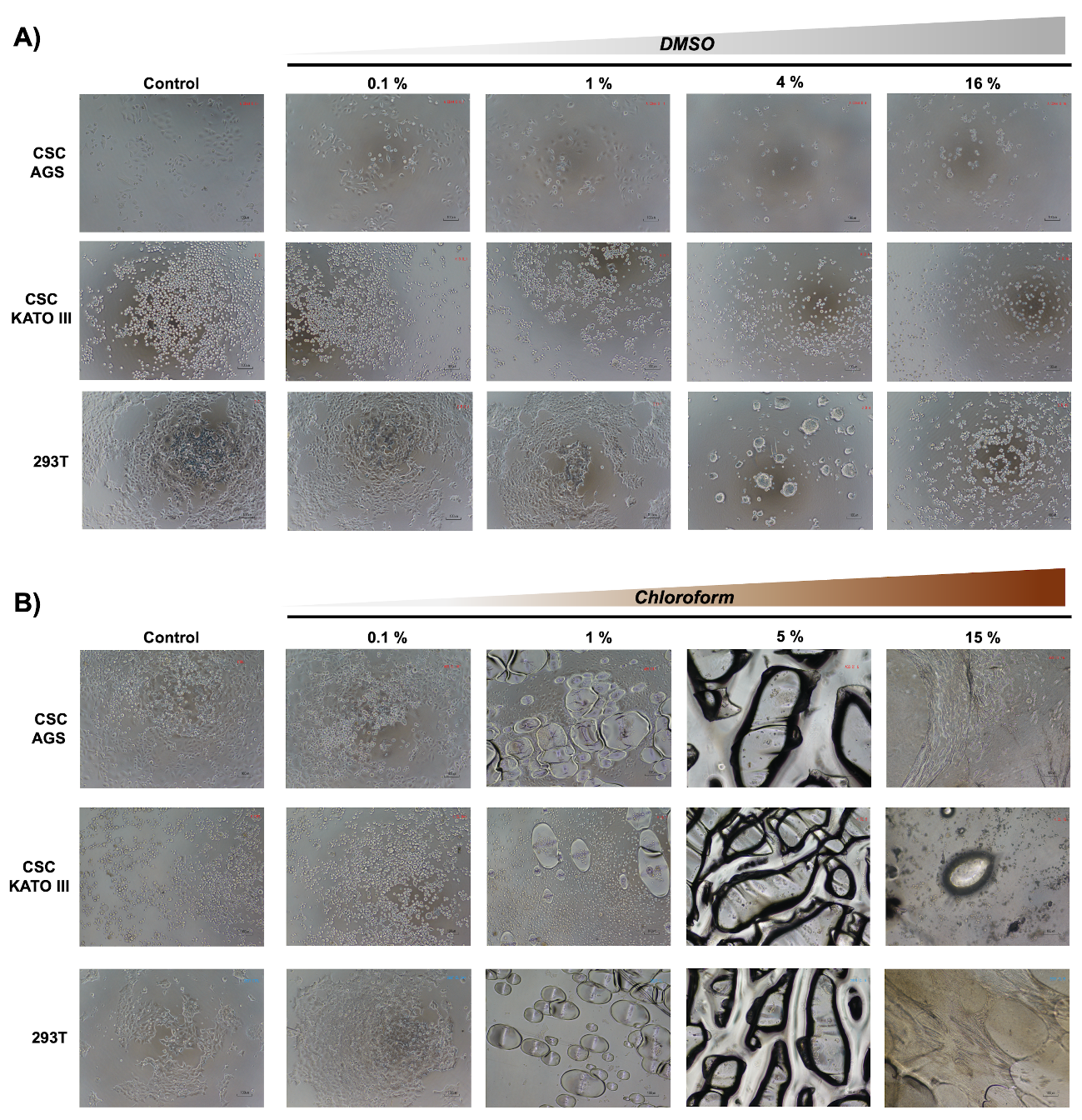


Supplementary Fig. 2. Morphological effects of DMSO and chloroform on CSCs and non-cancerous cells. Representative phase-contrast microscopy images of AGS CSCs, KATO III CSCs and 293T cells treated for 48 h with increasing concentrations of DMSO (A) or chloroform (B). Images were captured at 20X magnification; scale bar = 100 µm.


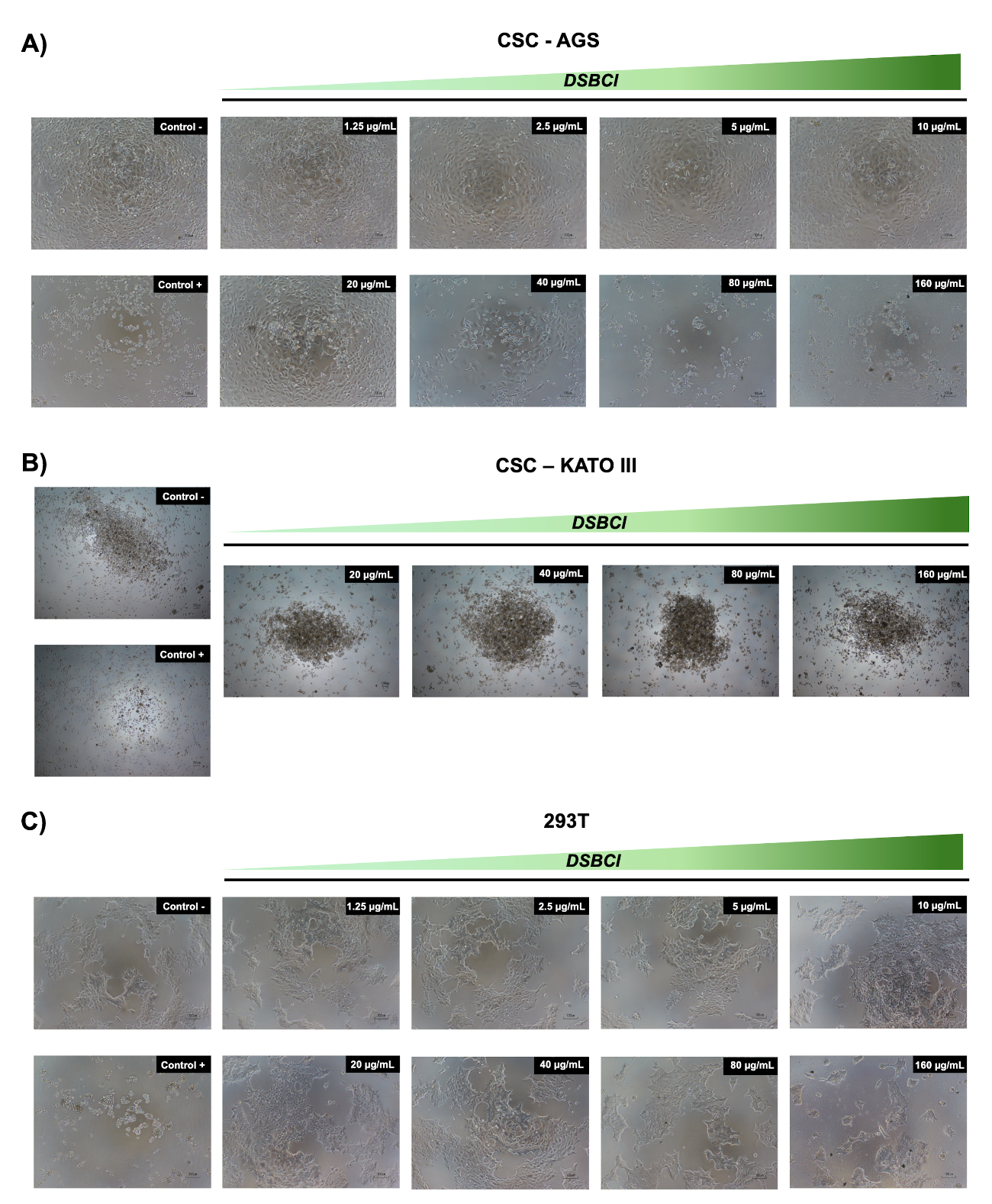


Supplementary Fig. 3. Dose-dependent morphological changes induced by DSBCl in CSCs and non-cancerous cells. AGS CSCs (A) and KATO III CSCs (B), and 293T cells (C) were treated for 48 h with increasing concentrations of the chloroform fraction of Dracontium spruceanum (DSBCl). Images show representative changes in cell morphology compared to cells treated with 0.1% DMSO in culture medium (Control −) and cells treated with 15% DMSO in culture medium (Control +). CSCs showed altered cell density and loss of adherence at higher concentrations, while 293T cells exhibited moderate changes. Images acquired at 20X magnification; scale bar = 100 µm.


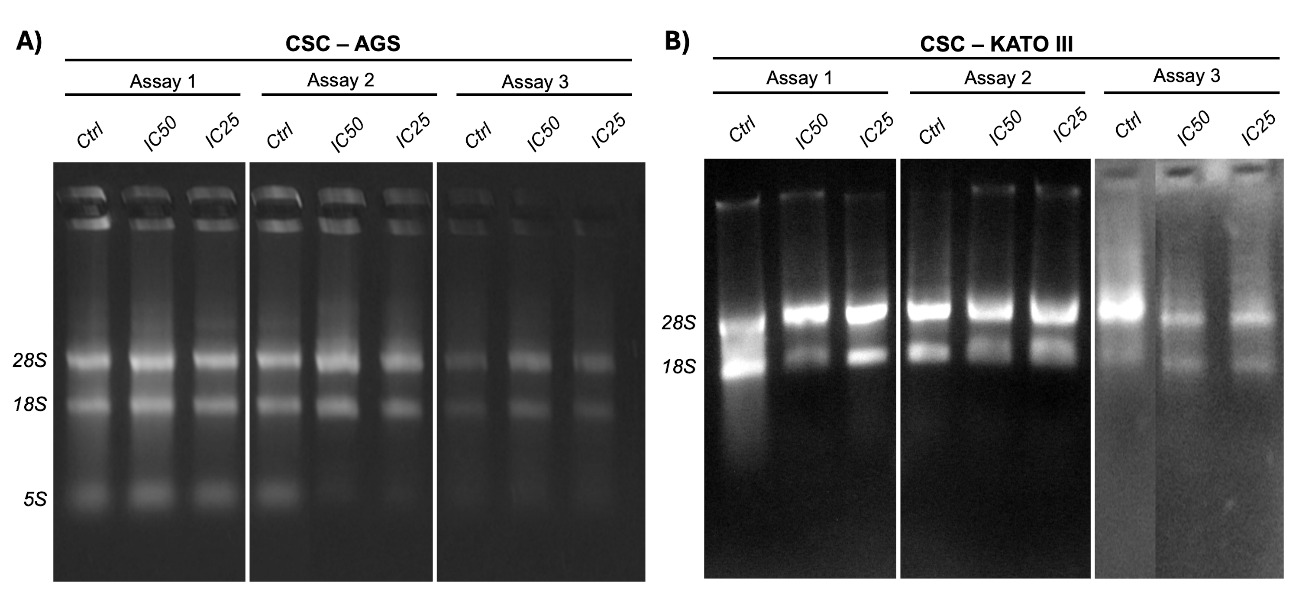


Supplementary Fig. 4. Evaluation of RNA integrity in CSCs following DSBCl treatment. Total RNA extracted from AGS CSCs (A) and KATO III CSCs (B) after 48 h of treatment with IC₂₅ and IC₅₀ of DSBCl. Representative images of three independent assays (Assay 1–3) are shown for each cell line. The bands corresponding to 28S, 18S, and 5S ribosomal RNA are indicated. The final figure was assembled from representative gel lanes captured in separate images, all obtained under the same electrophoretic conditions, to illustrate RNA quality under each treatment condition.


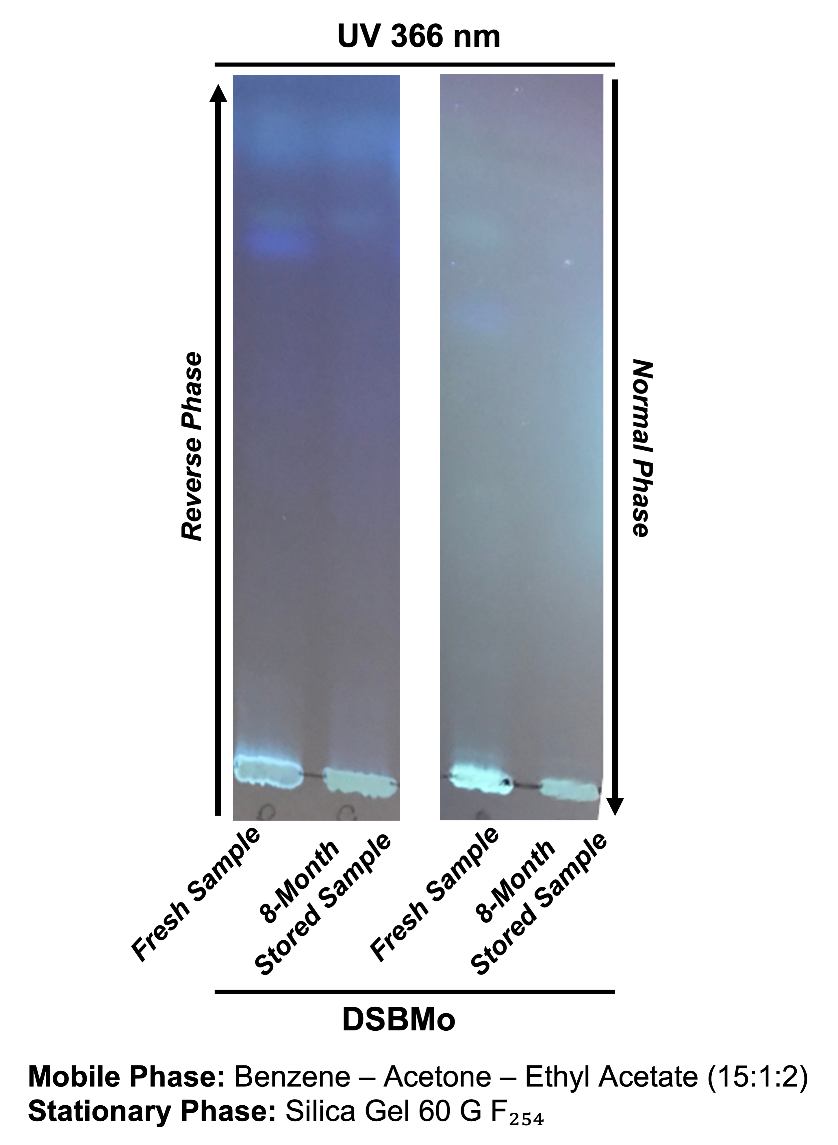


Supplementary Fig 5. Thin-layer chromatography (TLC) analysis of DSBMo to assess phytochemical stability over storage time. TLC was performed on freshly prepared and 8-month stored samples of the methanolic extract from *Dracontium spruceanum* (DSBMo) and visualized under UV light at 366 nm. Two chromatographic systems were used: reverse-phase (left panel) and normal-phase (right panel). The mobile phase was composed of benzene:acetone:ethyl acetate (15:1:2), and the stationary phase was silica gel 60 G F₂₅₄. The similar banding patterns observed indicate the preservation of major UV-detectable compounds after storage under controlled conditions.

Supplementary table 1. Primer sequences, expected amplicon sizes, and annealing temperatures used for PCR

| **Gene** | **Sequence (5` --> 3`)** | **Mature mRNA Amplicon (bp)** | **DNA Amplicon (pb)** | **Teoric Ta (°C)** | **Experimental Ta (°C)** |
| --- | --- | --- | --- | --- | --- |
| *NANOG* | F: AATACCTCAGCCTCCAGCAGATG | 148 | 1392 | 58 | 60 |
|  | R: TGCGTCACACCATTGCTATTCTTC |  |  |  |  |
| *OCT 4* | F: GTGCCGTGAAGCTGGAGAA | 192 | - | 60 | 60 |
|  | R: TGGTCGTTTGGCTGAATACCTT |  |  |  |  |
| *MYC* | F: GTCAAGAGGCGAACACACAAC | 162 | 162 | 61.8 | 60 |
|  | R: TTGGACGGACAGGATGTATGC |  |  |  |  |
| *KLF4* | F: CCCACATGAAGCGACTTCCC | 170 | 170 | 62 | 60 |
|  | R: CAGGTCCAGGAGATCGTTGAA |  |  |  |  |
| *BCL2L1* | F: GAGCTGGTGGTTGACTTTCTC | 119 | 119 | 61 | 60 |
|  | R: TCCATCTCCGATTCAGTCCCT |  |  |  |  |
| *ABCC2* | F: CCCTGCTGTTCGATATACCAATC | 131 | 1322 | 60.5 | 60 |
|  | R: TCGAGAGAATCCAGAATAGGGAC |  |  |  |  |
| *BAX* | F: CCCGAGAGGTCTTTTTCCGAG | 155 | - | 62 | 60 |
|  | R: CCAGCCCATGATGGTTCTGAT |  |  |  |  |
| *ID1* | F: GGCTGTTACTCACGCCTCAAG | 112 | 112 | 61 | 60 |
|  | R: CCAACTGAAGGTCCCTGATGTAG |  |  |  |  |
| *KLF17* | F: CCCCTCAGCAAGAGATGACG | 81 | 81 | 61.5 | 60 |
|  | R: GCCTGGCTACCCTTGGAAT |  |  |  |  |
| *RPL29* | F: CAGTCCCGAAAATGGCACAGA | 200 | 1115 | 62 | 60 |
|  | R: GGCTTTACGAGGGCCTTGATA |  |  |  |  |
| *GAPDH* | F: ACAACTTTGGTATCGTGGAAGG | 101 | 292 | 61 | 60 |
|  | R: GCCATCACGCCACAGTTTC |  |  |  |  |
| *PKG1* | F: GGAGAACCTCCGCTTTCAT | 78 | 78/3229 | 60 | 60 |
|  | R: GCTGGCTCGGCTTTAACC |  |  |  |  |
| *ACTB* | F: CTCACCATGGATGATGATATCGC | 167 | 301 | 58.7 | 60 |
|  | R: ACATAGGAATCCTTCTGACCCA |  |  |  |  |
| *TBP* | F: TTGGGTTTTCCAGCTAAGTTCT | 140 | 1770 | 60 | 60 |
|  | R: CCAGGAAATAACTCTGGCTCA |  |  |  |  |
| *B2M* | F: TTCTGGCCTGGAGGCTATC | 86 | 3895 | 60 | 60 |
|  | R: TCAGGAAATTTGACTTTCCATTC |  |  |  |  |
